# Measurement of the Physical Properties during Laparoscopic Surgery Performed on Pigs by Using Forceps with Pressure Sensors

**DOI:** 10.1155/2015/495308

**Published:** 2015-02-17

**Authors:** Hiroyuki Yamanaka, Kazuhide Makiyama, Kimito Osaka, Manabu Nagasaka, Masato Ogata, Takahiro Yamada, Yoshinobu Kubota

**Affiliations:** ^1^Department of Urology, Yokohama City University, Yokohama 236-0004, Japan; ^2^Mitsubishi Precision Co., Ltd., Kamakura 247-8505, Japan; ^3^Graduate School of Environment and Information Sciences, Yokohama National University, Yokohama 240-8501, Japan

## Abstract

*Objectives*. Here we developed a unique training system, a patient specific virtual reality simulator, for laparoscopic renal surgery. To develop the simulator, it was important to first identify the physical properties of the organ. *Methods*. We recorded the force measured during laparoscopic surgery performed on pigs by using forceps with pressure sensors. Several sensors, including strain gauges, accelerometers, and a potentiometer, are attached to the forceps. *Results*. Throughout the experiment, we measured the reaction force in response to the forceps movement in real time. *Conclusions*. The experiment showed the possibility of digitizing these physical properties in humans as well.

## 1. Background

Laparoscopic surgery has become an increasingly common practice in recent years because it is less invasive than traditional methods [1]. However, surgeons must be highly skilled to perform laparoscopic surgery since it is one of the most difficult surgical techniques to learn and involves a steep learning curve [1]. Surgeons must acquire laparoscopic skills before performing laparoscopy in the operating room [2–6].

Like flight and driving simulators, laparoscopy simulators must provide a virtual yet accurate simulation of the task at hand. For example, in a flight simulator, the view and sounds in the cockpit are very real. Some flight simulators also demonstrate acceleration.

**Figure 1:**
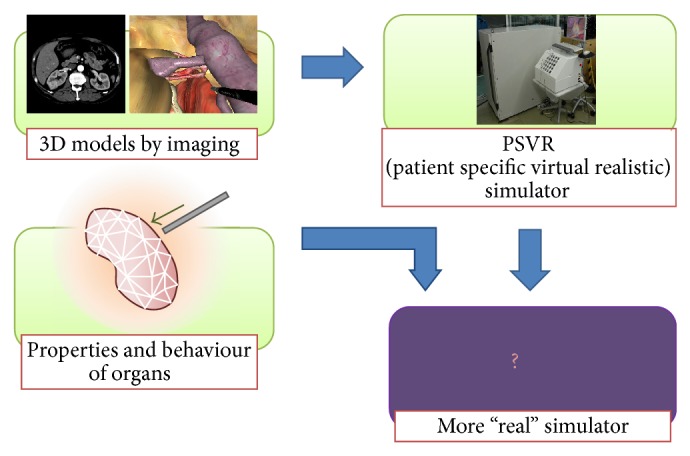
To make our simulator performance more “real,” we considered the importance of the subjective sensation that surgeons feel while using it.

The latest laparoscopy simulators can reproduce an entire laparoscopic surgery [7–9]. Some training systems that simulate surgical processes are commercially available. Such systems are useful for basic training. They demonstrated initial construct validity regarding force and position sensing and capable of detecting differences between novices and experts in a laparoscopic suturing task with respect to force and position [10, 11]. However, they do not provide surgeons with the necessary experience to respond to specific conditions in individual patients. Therefore, we have developed a unique training system, called a PSVR type simulator, for laparoscopic surgery [12–15]. Using data specific to each individual patient, this system facilitates “rehearsal” operations for surgeons. We use multislice CT imaging technology in laparoscopic surgery, and CT images of individual patients who are scheduled to undergo surgery are transferred into the simulation system (Figure 1). Each patient's specific organ volume data are extracted by our simulator to allow surgeons to perform a preoperative “rehearsal.” Some PSVR simulators are reportedly in commercial use, but no PSVR simulator is currently available in the field of urology [16, 17].

In an effort to make our simulator performance more “real,” we considered the importance of the subjective sensation that surgeons feel while using it. Other commercial surgical simulators are validated by the adjustment of the physical parameters of the deformable model and reliance on the surgeon's subjective sensation [11, 16, 17]. Those simulators might present a reaction force to the surgeon that is similar to a certain degree to that of a real operation. However, an evaluation that relies only on the surgeon's sensation is subjective and lacks objectivity. Our mechanical model, based on the corotated finite element method [15, 18], has not been numerically verified with real soft tissue during surgery.

To resolve these issues, the collection of numerical data such as the forceps reaction force, grabbing angle, and moving speed is necessary. Unfortunately, no such measurement data currently exist. Therefore, as a first step, we recorded the force measured during laparoscopic surgery performed on pigs by using forceps with pressure sensors. During the experiment, we measured the reaction force and gripping force of the membrane, kidney, liver, and vessels.

## 2. Materials and Methods

**Figure 2:**
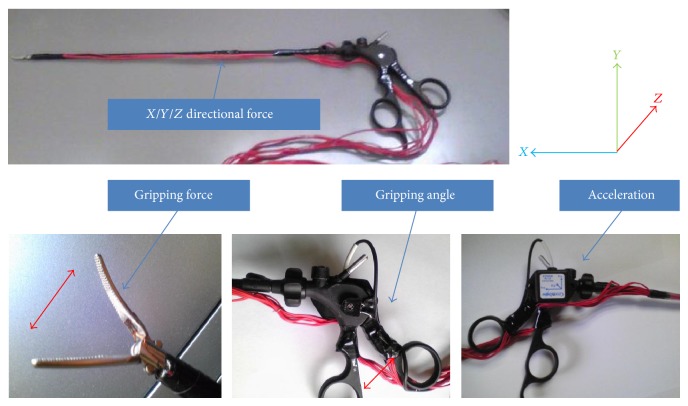
The forceps we developed.

We developed a multimodal measuring device that interferes very little with the surgeon's movements. The system was developed through collaboration among Yokohama City University, Mitsubishi Precision, and Yokohama National University (our homepage: http://www-user.yokohama-cu.ac.jp/~urology/kenkyu/surgicalsimulatorindex.html). The measuring device is illustrated in Figure 2 and Table 1.

Figure 2 shows the forceps and the sensor wires. The running sensor wires are packed inside of the instrument and we use special guide when we insert the forceps to the trocar. So the forceps is free from contact or damage and we can observe the accurate measurements.

**Figure 3:**
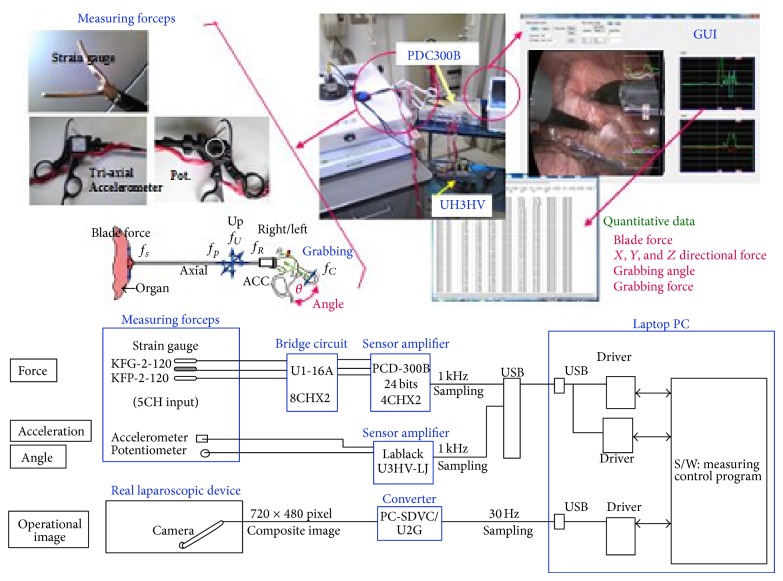
The system we developed to detect the sensation of the forceps.

The block diagram is presented in Figure 3. Several sensors (e.g., strain gauges, accelerometers, and a potentiometer) are attached to the forceps to measure the *X*/*Y*/*Z* directional forces, blade force, grabbing force, grabbing angle, and acceleration. All of these parameters are measured over 0.1 ms and stored on a hard disk drive for later analysis. To synchronize the acquired physical quantities, such as the reaction force, with the corresponding surgical operations, we adopted a method to overlap a plotted graphical image of quantitative data.

We calibrated the system and then checked the control. Under this condition, we performed a laparoscopic nephrectomy on a pig by using multimodal measuring forceps.

We performed the laparoscopic nephrectomy as follows: (1) we made an incision in the peritoneum and displaced the colon; (2) we exposed the ureter and renal artery and vein; (3) we exposed the renal capsule; (4(A)) we ruptured the kidney by using forceps; (5) we sutured the ruptured kidney by using a surgical needle; and (6) we dissected the renal artery and vein after ligation.

During and after the nephrectomy, we measured the reaction force of the organs as follows: (B) we gripped the gonadal vein; (C) we pulled on the renal artery; (D) we pulled on the renal vein; (E) we pulled on the ureter; (F) we gripped the liver softly; (G) we gripped the liver strongly; (H) we gripped and pulled the liver; (I) we gripped the kidney softly; (J) we gripped the kidney strongly; and (K) we gripped and pulled the kidney.

The forceps used for the measurements are fragile, so we used normal forceps or energy devices when not taking the measurements. We collected these measurements three or four times to obtain an appropriate average reaction force as well as the max reaction force. This experiment was examined by ethical committee of Yokohama City University and accepted (Research number B100902033: the measurement of transformation and physical properties of organ by the external force). The experiment was performed under the ethical consideration.

## 3. Results

The results are shown in Table 2. The force (N) is determined as the maximum force during the experiment.

**Figure 4:**
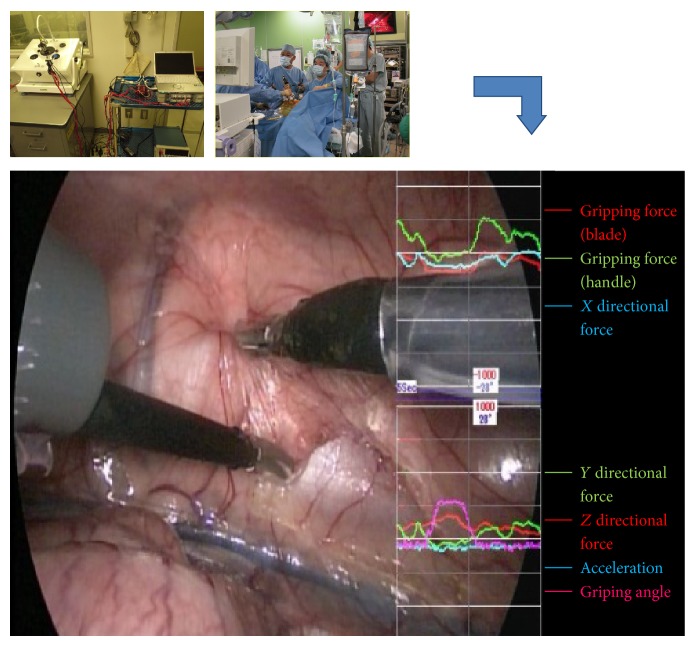
To synchronize the acquired physical quantities, such as the reaction force, with the corresponding surgical operations, we adopted a method to overlap a plotted graphical image of quantitative data.

**Figure 5:**
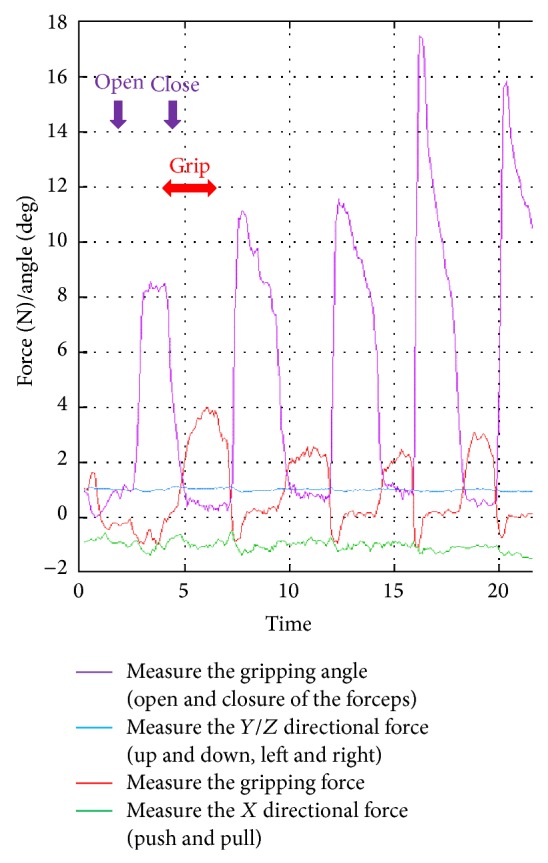
The green line represents the *X* directional force, the red line represents the grabbing force, and the blue line represents the sum of the *Y* and *Z* directional forces. The angles of the blade are shown as a purple line, and the measurements are synchronized with the blade opening and closing.

The sample results presented in Figures 4 and 5 show the reaction force on the blade and the corresponding grabbing force. The green line represents the *X* directional force, the red line represents the grabbing force, and the blue line represents the sum of the *Y* and *Z* directional forces. The angles of the blade are shown as a purple line, and the measurements are synchronized with the blade opening and closing, suggesting that the experimental data interference is small. 

**Figure 6:**
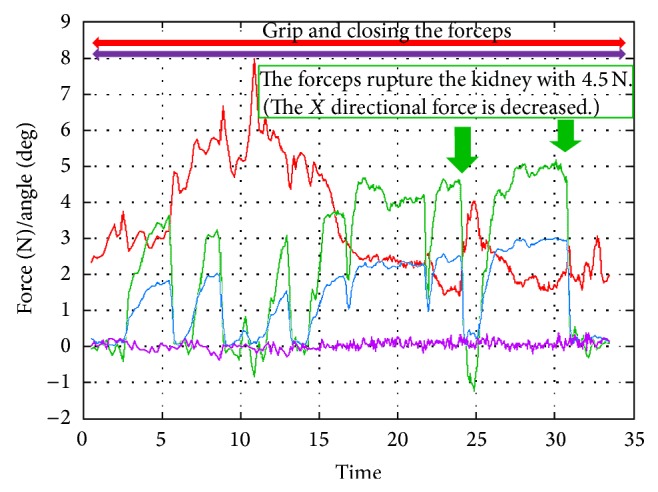
Experiment (A), rupture the kidney.

*(A) Rupture the Kidney*. The surgeon tried to rupture the kidney by using the tip of the closed forceps. When the tip of the blade pushed on the kidney, the forceps received a reaction force (green line). When we pushed kidney with 4.5 N, it ruptured. As such, we assume that the reaction force required to rupture the kidney was 4.5 N from the *X* directional force with the tip of the closed forceps (Figure 6). 

**Figure 7:**
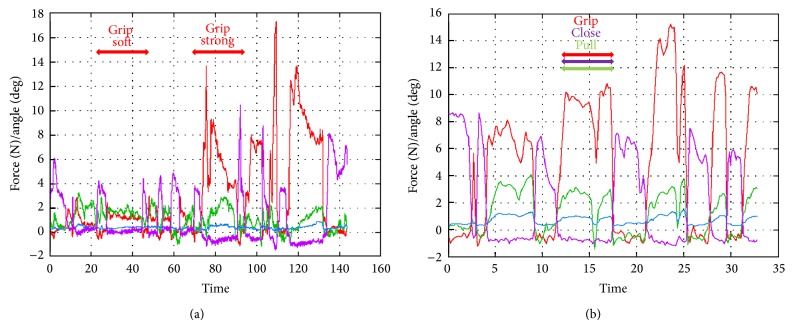
Experiment (B), grip the gonadal vein.

*(B) Grip the Gonadal Vein*. When the surgeon gripped the gonadal vein softly and strongly, the gripping force reached 2 and 17 N, respectively. A total of 8–15 N of gripping force and 3 N of pulling force were required (Figure 7).

**Figure 8:**
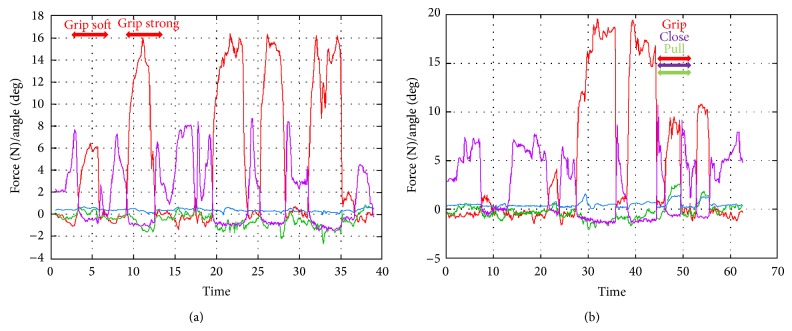
Experiment (C), grip and pull on the renal artery.

*(C) Grip and Pull on the Renal Artery*. When we gripped the renal artery softly and strongly to stop the blood flow, the gripping force reached 6 and 16 N, respectively. When the surgeon pulled and gripped the renal artery to stretch it, 8–10 N of gripping force and 3 N of pulling force were needed (Figure 8).

**Figure 9:**
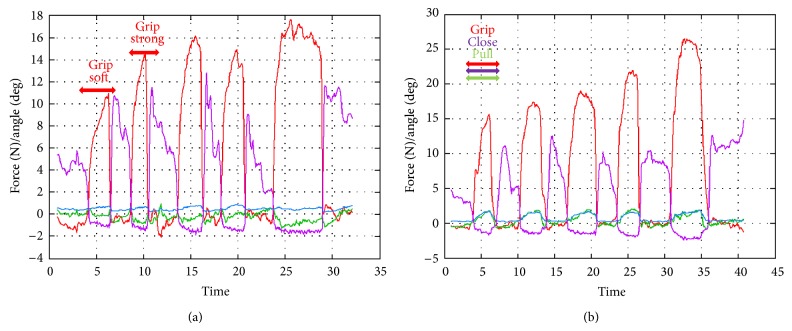
Experiment (D), grip and pull on the renal vein.

*(D) Grip and Pull on the Renal Vein*. When we gripped the renal vein softly and strongly to stop the blood flow, the gripping forces reached 10 and 16 N, respectively. We gripped and pulled the renal vein, and just before it tore off, it needed 15–20 N of gripping force and 2 N of pulling force (Figure 9).

**Figure 10:**
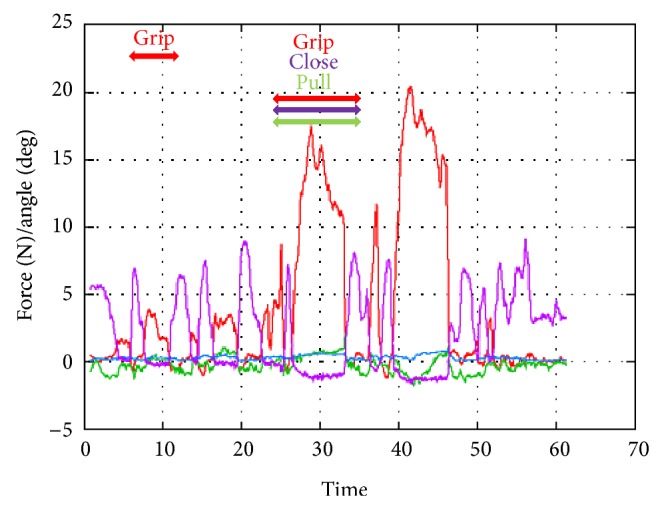
Experiment (E), grip and pull the ureter.

*(E) Grip and Pull the Ureter*. The ureter required 10–16 N of gripping force and 2 N of pulling force to be torn off (Figure 10).

**Figure 11:**
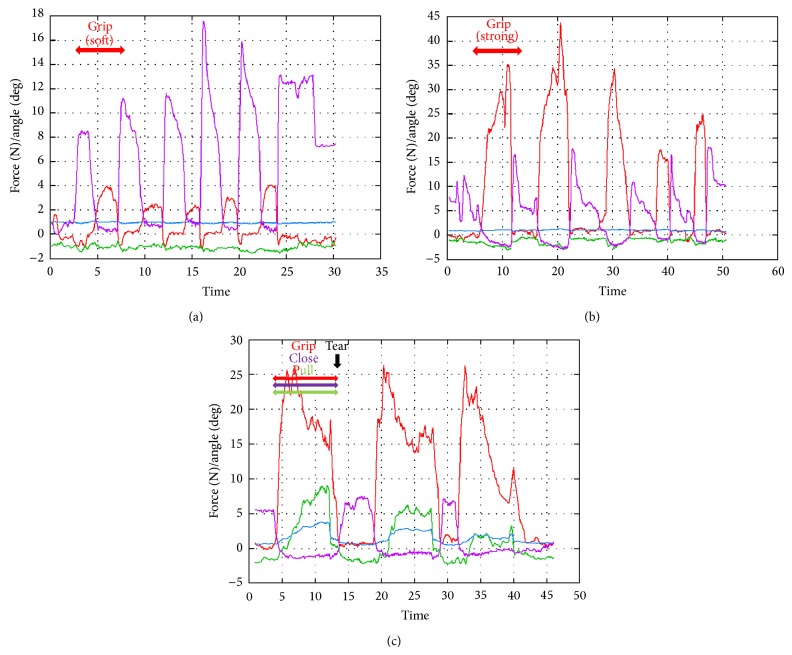
Experiment (F), grip liver softly; (G) grip the liver strongly; (H) grip and pull on the liver.

*(F) Grip of Liver Softly; (G) Grip the Liver Strongly; (H) Grip and Pull on the Liver*. When the surgeon gripped the liver softly, the forceps received 2–4 N of force. On the other hand, when the surgeon gripped the liver strongly, the forceps received 18–42 N of force. Moreover, he could tear off the liver by gripping the forceps with 26 N of force and pulling with 9 N of force (Figure 11). 

**Figure 12:**
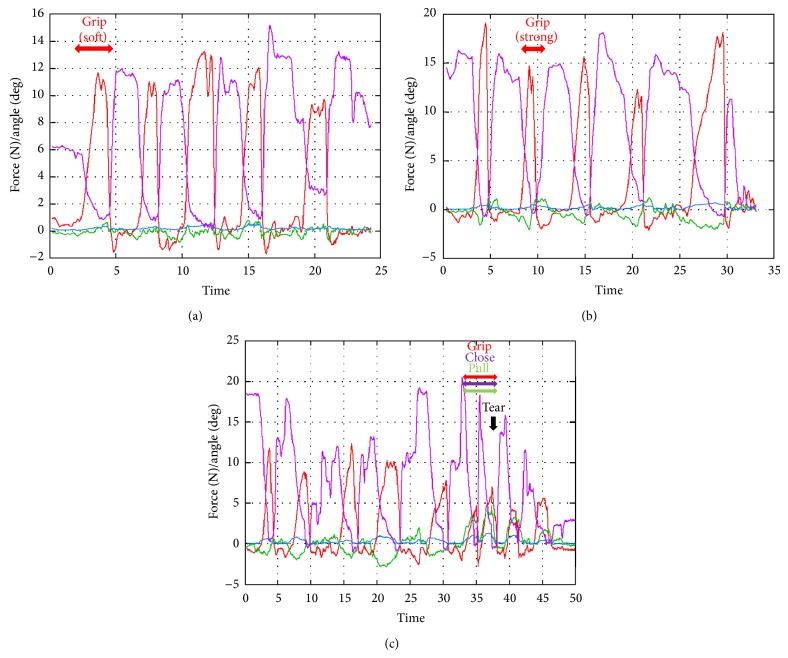
Experiment (I), grip the kidney softly; (J) grip the kidney strongly; (K) grip and pull on the kidney.

*(I) Grip the Kidney Softly; (J) Grip the Kidney Strongly; (K) Grip and Pull on the Kidney*. When the surgeon gripped the liver softly, the forceps received 10–13 N of force. On the other hand, when the surgeon gripped the liver strongly, the forceps received 11–18 N of force. Moreover, the liver was torn off with 12 N of gripping force and 4 N of pulling force (Figure 12).

## 4. Discussion

When the surgeon pulled on the renal artery, renal vein, gonadal vein, and ureter just before tearing them off, the forceps received large gripping forces. As such, we believe the following: the renal artery bears 10 N of gripping force and 3 N of pulling force; the renal vein bears 20 N of gripping force and 2 N of pulling force; the gonadal vain bears 15 N of gripping force and 3 N of pulling force; and the ureter bears 16 N of gripping force and 2 N of pulling force. These findings show that if a surgeon grips and pulls the renal artery, renal vein, gonadal vein, or ureter rather roughly, each can resist the surgical force to some extent. To stop the blood flow during an operation, we must know blood pressure and vessel properties. These properties may be changed by patient age or history [19], so we need to perform more experiments.

We gripped and pulled the liver and kidney, to measure their properties and determine the force at which they tear off. The edge of the liver is flat, while that of the kidney is round, so the surgeon can easily grip the liver with less gripping force. However, more force is needed to tear it off. This shows that the liver is solid compared with the kidney.

Figure 6 shows that, to rupture the kidney, for example, the surgeon gripped the forceps strongly to push the forceps into the kidney. He initially gripped the forceps too strongly. The red line demonstrated 8 N of force. After he got used to handling the forceps and was familiar with the moderate amount of power needed to grip and push the kidney, the gripping force tended to decrease (red line demonstrated 2 N of force). The gripping force increased to 4 N the moment of rupture, as the surgeon gripped the forceps strongly because he noticed the sudden shock when the kidney was ruptured.

These findings demonstrated that the operator tends to grip the forceps strongly when he notices the sudden shock of the forceps. As such, it may be possible to use gripping force to detect an operator's skill level since beginners tend to grip the forceps strongly. The gripping force as well as the moving speed or acceleration may be signs of operator proficiency. Thus, surgical techniques between operators can be evaluated and scored. Indeed, Yoshida et al. examined forceps forces and application time, and their results suggest that experts should try to keep the instrument tip within the operative field [20]. Trejos et al. also show that force-based metrics were able to provide stronger correlations with experience than those found with task completion time or position based metrics [11].

Many experiments have tried to detect the properties of the organs or materials using operative devices or other kinds of equipment [10, 11, 19–24]. While many other experiments have been performed in a Dry-Box with a metal cylinder, our experiment was performed in a real operative environment and with real forceps that can measure gripping force, directional force, and the blade's angle. Here we used Maryland-type dissecting forceps. If the shape of the blade is changed, the *X* directional force or gripping force may be changed. In fact, it has been shown that 15 N of force is needed to destroy liver tissue by using a rubber plate [21], whereas our experimental data showed that 30–42 N is required to destroy liver tissue and 26 N is required to tear it off.

Some experiments have been performed with real forceps and dead pig organs [20, 23]. These gripping or directional forces are assumed by the gripping of the handle's angle and the force or sensor attached to the forceps shaft or handle. On the other hand, our dynamic sensors are attached to not only the shaft and handle but also the tip of the blade. Since our forceps directly detect the gripping force from the shaft and the directional force from the blade, our results are free of functional noise. Moreover, our forceps can be used in a real operative environment and can be inserted with the trocar in laparoscopic surgery, making it technically possible to perform such experiments in humans. However, there are associated ethical problems. Of course, we should also perform this kind of experiment in a Dry-Box to fine-tune our simulator and complement the shortage of actual experiments. We never have an intention to perform our experiment on living human body but we may perform this kind of experiment in a Dry-Box with use of extracted organs by carcinoma with patient's agreement. In that case, we will compare measurement data of pig with human and improve the simulator's sensors. Certainly, this experiment does not improve our “patient specific” simulation. However, this acquired data improves general sensation or action of our simulator and is efficacious for good compulsory training programs. As such we believe that our result is realistic and will lead to a more “real” simulation experience.

## 5. Conclusions

Here we recorded the force measured during laparoscopic surgery performed on pigs by using forceps with pressure sensors and performed a laparoscopic nephrectomy on a pig by using multimodal measuring forceps. The forceps measured the *X*/*Y*/*Z* directional forces, blade force, grabbing force, and grabbing angle in real operative situations within a pig. The experiment showed the possibility of digitizing the physical properties in humans as well.

## Figures and Tables

**Table 1:** The equipment we used.

	Name	Type
1	Forceps	K33310MD, KARL STORZ
2	Controller & amplifier	PDC300B, Kyowa
3	Controller & amplifier	U3HV, Lab Jack
4	Bridge	U1-16A, Kyowa
5	Laptop PC	CF-S10, Panasonic, Windows 7 64 bits
6	Acc.	CXL17LF3, Crossbow
7	Converter	PC-SDVC/U2G, BUFFALO

**Table 2:** The results of the experiments.

	Pulling/*X* directional force (N)	Gripping force (N)
(A) Rupture the kidney	5	4
(B) Grip the gonadal vein	—	2 (soft)–17 (strong)
(B) Pull on the gonadal vein	3	8–15
(C) Pull on the renal artery	3	15–20
(D) Pull on the renal vein	2	15–20
(E) Pull on the ureter	2	10–16
(F) Grip the liver softly	—	2–4
(G) Grip the liver strongly	—	18–42
(H) Grip and pull on the liver	9	26
(I) Grip the kidney softly	—	10–13
(J) Grip the kidney strongly	—	11–18
(K) Grip and pull on the kidney	4	12

## Abbreviations and Acronyms

CT: Computed tomography
PSVR: Patient specific virtual reality.
